# 180-Nucleotide Duplication in the G Gene of *Human metapneumovirus* A2b Subgroup Strains Circulating in Yokohama City, Japan, since 2014

**DOI:** 10.3389/fmicb.2017.00402

**Published:** 2017-03-14

**Authors:** Miwako Saikusa, Chiharu Kawakami, Naganori Nao, Makoto Takeda, Shuzo Usuku, Tadayoshi Sasao, Kimiko Nishimoto, Takahiro Toyozawa

**Affiliations:** ^1^Yokohama City Institute of Public HealthYokohama, Japan; ^2^Department of Virology III, National Institute of Infectious DiseasesMusashimurayama, Japan; ^3^Yokohama City Public Health CenterYokohama, Japan

**Keywords:** *Human metapneumovirus*, molecular epidemiology, G gene, duplication, surveillance

## Abstract

*Human metapneumovirus* (HMPV), a member of the family *Paramyxoviridae*, was first isolated in 2001. Seroepidemiological studies have shown that HMPV has been a major etiological agent of acute respiratory infections in humans for more than 50 years. Molecular epidemiological, genetic, and antigenetic evolutionary studies of HMPV will strengthen our understanding of the epidemic behavior of the virus and provide valuable insight for the control of HMPV and the development of vaccines and antiviral drugs against HMPV infection. In this study, the nucleotide sequence of and genetic variations in the G gene were analyzed in HMPV strains prevalent in Yokohama City, in the Kanto area, Japan, between January 2013 and June 2016. As a part of the National Epidemiological Surveillance of Infectious Diseases, Japan, 1308 clinical specimens (throat swabs, nasal swabs, nasal secretions, and nasal aspirate fluids) collected at 24 hospitals or clinics in Yokohama City were screened for 15 major respiratory viruses with a multiplex reverse transcription–PCR assay. HMPV was detected in 91 specimens, accounting for 7.0% of the total specimens, and the nucleotide sequences of the G genes of 84 HMPV strains were determined. Among these 84 strains, 6, 43, 10, and 25 strains were classified into subgroups A2a, A2b, B1, and B2, respectively. Approximately half the HMPV A2b subgroup strains detected since 2014 had a 180-nucleotide duplication (180nt-dup) in the G gene and clustered on a phylogenic tree with four classical 180nt-dup-lacking HMPV A2b strains prevalent between 2014 and 2015. The 180nt-dup causes a 60-amino-acid duplication (60aa-dup) in the G protein, creating 23–25 additional potential acceptor sites for O-linked sugars. Our data suggest that 180nt-dup occurred between 2011 and 2013 and that HMPV A2b strains with 180nt-dup (A2b_180nt-dup_ HMPV) became major epidemic strains within 3 years. The detailed mechanism by which the A2b_180nt-dup_ HMPV strains gained an advantage that allowed their efficient spread in the community and the effects of 60aa-dup on HMPV virulence must be clarified.

## Introduction

The aim of this study was to strengthen our understanding of the epidemic behavior of *Human metapneumovirus* (HMPV) by providing virological data on the distribution patterns of HMPV. HMPV was first isolated in 2001 from young children suffering acute respiratory infections (ARIs), and was classified in the subfamily *Pneumovirinae* in the family *Paramyxoviridae* (van den Hoogen et al., 2001). Seroepidemiological studies have shown that the virus has been circulating globally for more than 50 years (van den Hoogen et al., 2001). HMPV is a major cause of upper and lower ARIs in infants and children (Collins and Karron, 2013), and also causes severe ARIs in aged adults and patients with underlying diseases (Boivin et al., 2002; Stockton et al., 2002; Falsey et al., 2003). Most children experience their first infection with HMPV before 5 years of age, but this infection does not provide lifelong immunity, and reinfection occurs frequently (van den Hoogen et al., 2001; Ebihara et al., 2004). In Japan, epidemics of HMPV have occurred between January and June, and especially in March and April (Kaida et al., 2006; Mizuta et al., 2010, 2013; Nakamura et al., 2013).

HMPV has a non-segmented negative-strand RNA genome of ∼13 kb. The HMPV genome contains eight genes in the order: 3′-N–P–M–F–M2–SH–G–L-5′ (Collins and Karron, 2013). The virus has three types of transmembrane viral proteins in its envelope, the fusion (F) protein, small hydrophobic (SH) protein, and glycoprotein (G protein), which are encoded by the F, SH, and G genes, respectively (Collins and Karron, 2013). The F protein is responsible for viral attachment and membrane fusion and is essential for viral infectivity (Biacchesi et al., 2004, 2005). Cell-surface integrins and glycosaminoglycans play roles in viral attachment and membrane fusion, mediated by the F protein (Cseke et al., 2009; Chang et al., 2012; Cox et al., 2012, 2015). The SH protein has properties consistent with those of viroporins and modulates the viral fusogenic activity (Masante et al., 2014). The G proteins of some lineages of HMPV also bind to glycosaminoglycans and contribute to HMPV infection (Thammawat et al., 2008; Adamson et al., 2012, 2013). The short cytoplasmic domain of the G protein inhibits the RIG-I-dependent signaling pathways (Bao et al., 2008). Despite these roles, the G and SH proteins are not essential for viral infectivity, but function as virulence factors (Biacchesi et al., 2004, 2005). Therefore, the G protein can be targeted in the development of antiviral drugs. The F protein is highly immunogenic and induces protective immunity, whereas the G and SH proteins are poorly immunogenic (Skiadopoulos et al., 2006).

The G protein is the most variable of the HMPV proteins, and mutations predominantly accumulate in the extracellular domain (Peret et al., 2004). The G protein has multiple potential glycosylation sites, and glycosylation can modify its immunogenicity (Liu et al., 2007). An evolutionary analysis of HMPV suggested that selective pressure is exerted on the G protein by the host’s adaptive immunity (Gaunt et al., 2011). HMPV is divided to two groups, A and B, based on variations in its nucleotide sequence and its reactivity to monoclonal antibodies (van den Hoogen et al., 2004). The G gene has the most variable nucleotide sequence, and each viral group is further divided to two subgroups, A1 and A2 in group A, and B1 and B2 in group B, based mainly on the variations in the G gene (Biacchesi et al., 2003; van den Hoogen et al., 2004). Further detailed analyses of HMPV strains have also suggested two clades in the A2 subgroup, A2a and A2b (Huck et al., 2006). These different subgroups of HMPV have been detected in varying proportions in different countries and regions. In this study, we identified the genetic variations in the G gene of the HMPV strains prevalent in Yokohama City, in the Kanto area, Japan, between January 2013 and June 2016. Our data demonstrate a 180-nucleotide (nt) duplication (180nt-dup) in the G gene of HMPV and suggest that 180nt-dup occurred between 2011 and 2013. The HMPV A2b strains containing 180nt-dup (A2b_180nt-dup_ HMPV strains) became major epidemic strains within 3 years, possibly overwhelming the classical A2b HMPV strains.

## Materials and Methods

### Clinical Samples and HMPV Detection

In Yokohama City between January 2013 and June 2016, 1308 clinical specimens (throat swabs, nasal swabs, nasal secretions, and nasal aspirate fluids) were collected from patients suffering upper or lower ARIs in 16 sentinel hospitals and clinics (eight pediatric clinics, four internal medicine clinics, and four hospitals) participating in the National Epidemiological Surveillance of Infectious Diseases (NESID), instituted by the Infectious Diseases Control Law in Japan, and in eight other medical institutions (one pediatric clinic and seven hospitals) (Anon, 2010). There were 113 hospitals and 2962 clinics in Yokohama City, and the population in the city was ∼3.7 million. Thus, the numbers of hospitals and clinics participated in this study were ∼9.7 and ∼0.4%, respectively, in the city. Before collecting the clinical specimens in which to analyze the viruses causing ARIs, the physicians at each medical institution obtained the informed consent of the patients or their guardians. The de-identified clinical specimens were sent to Yokohama City Institute of Public Health and subjected to multiplex RT–PCR with the Seeplex^®^ RV15 OneStep ACE Detection kit (Seegene, Seoul, South Korea), which identifies 15 major respiratory viruses. The clinical specimens that were positive for HMPV were analyzed further.

### RNA Extraction and RT–PCR

The RNAs were purified from clinical specimens (140 μl) using the QIAamp Viral RNA Mini Kit (Qiagen, Hilden, Germany), according to the manufacturer’s instruction, and dissolved in 60 μl of distilled water. The RNA in 5 μl of the purified RNA solution was reverse transcribed, and the G gene was amplified from the cDNA with RT–PCR using the PrimeScript II High Fidelity One Step RT–PCR Kit (TaKaRa Bio, Otsu, Japan) and a pair of G-gene-specific primers, SH7 (5′ primer) (van den Hoogen et al., 2004) and GR (3′ primer) (Banerjee et al., 2011). The reaction mixture was prepared in a 50 μl solution, and the reverse-transcription reaction was performed at 45°C for 10 min. The reverse transcriptase was inactivated at 94°C for 2 min. The G gene cDNA was amplified with PCR with 40 cycles of 10 s at 98°C, 15 s at 56°C, and 10 s at 68°C. The cDNA products were separated electrophoretically in 3% NuSieve^TM^ 3:1 Agarose gel in 0.5% TBE buffer. When no G-gene-specific band was detected or was only barely detectable, 5 μl of the RT–PCR product was subjected to semi nested PCR. In this assay, the reaction mixture was prepared in a 50 μl solution, and the RT–PCR products were denatured at 94°C for 2 min. The G gene was then PCR amplified with 40 cycles of 10 s at 98°C, 15 s at 50°C, and 30 s at 68°C with Tks Gflex^TM^ DNA Polymerase (TaKaRa Bio) and a pair of G-gene-specific primers, SH7 (5′ primer) and GNR2 (3′ primer). The sequence of the GNR2 primer was 5′-GGATTCATTAAGAGGATCCATTG-3′. The products of the semi nested PCR were separated electrophoretically to detect the G gene.

### Sequencing

The nucleotide sequences of the G gene of the HMPV strains were determined with direct sequencing. The amplified HMPV G genes were purified with the QIAquick PCR Purification Kit (Qiagen). The PCR products were subjected to cycle sequencing with the BigDye Terminator ver. 1.1 Cycle Sequencing Kit (Applied Biosystems, Foster City, CA, USA) and the SH7, GR, and GNR2 primers. The products of the cycle sequencing reactions were purified through Centri-Sep Spin Columns (Thermo Fisher Scientific), and the nucleotide sequences were determined with a 3500 Genetic Analyzer (Applied Biosystems). The nucleotide sequence data for the HMPV strains were aligned and edited with the BioEdit software (ver. 7.2.5) (Hall, 1999).

### Phylogenetic Analysis

A multiple-sequence alignment was constructed with the MAFFT software (ver. 7.304b) using the default settings (Katoh et al., 2002). Phylogenetic analyses were performed with the maximum likelihood method in the MEGA software (ver. 7.0.20) (Kumar et al., 2016), and the statistical significance of the tree topologies was tested with bootstrapping (50 replicates).

### N- and O-Glycosylation Site Analysis

Potential acceptor sites for *N-* and *O*-linked sugars were predicted with the NetNGlyc 1.0^1^ and NetOGlyc 3.1 programs^2^, respectively.

### Nucleotide Sequence Accession Numbers

The nucleotide sequence data reported in the present study were deposited in the DDBJ/EMBL/GenBank nucleotide sequence database under accession numbers LC192170–LC192253.

### Estimating the Evolutionary Rate of the HMPV G Gene

The overall rate of evolutionary change (nucleotide substitutions per site per year) in the HMPV G gene was estimated with the BEAST 2 program^3^, which uses a Bayesian Markov Chain Monte Carlo (MCMC) approach (BEAST 2: A Software Platform for Bayesian Evolutionary Analysis^4^). An aligned G gene sequence dataset from the 84 HMPV strains detected in Yokohama City from 2013 to 2016 was analyzed with the BEAST 2 program using a strict HKY model. The MCMC chain was run for a sufficient length of time to ensure convergence (all expected sample size values exceeded 200 with a 10% burn-in).

### Ethics Statement

These analyses have been done as a part of the National Epidemiological Surveillance of Infectious Diseases, Japan (NESID) as stipulated under the Infectious Diseases Control Law, and before collecting the clinical specimens, physicians in each medical institution obtained the informed consent of the patients or their guardians. The ethics committee of Yokohama City Institute of Public Health approved this study.

### Prediction of the Year in Which 180nt-dup Occurred in the HMPV Genome

The consensus nucleotide sequence was determined using both the first 180-nucleotide sequence (1st-180nts) and the second 180-nucleotide sequence (2nd-180nts) in the 15 A2b_180nt-dup_ HMPV strains (in total, 30 sequences of 180 nt) and was assumed to be the corresponding 180 nt of the hypothetical ancestral strain of the 15 A2b_180nt-dup_ HMPV strains. The number of nucleotide substitutions in the 1st- and 2nd-180nts of each A2b_180nt-dup_ HMPV strain was determined and compared with the corresponding 180 nt of the hypothetical ancestral strain, and the year in which 180nt-dup occurred in the HMPV genome was estimated based on the mean rate of nucleotide substitutions for the HMPV G gene.

## Results

### Nucleotide Sequence and Phylogenetic Analyses

**FIGURE 1:**
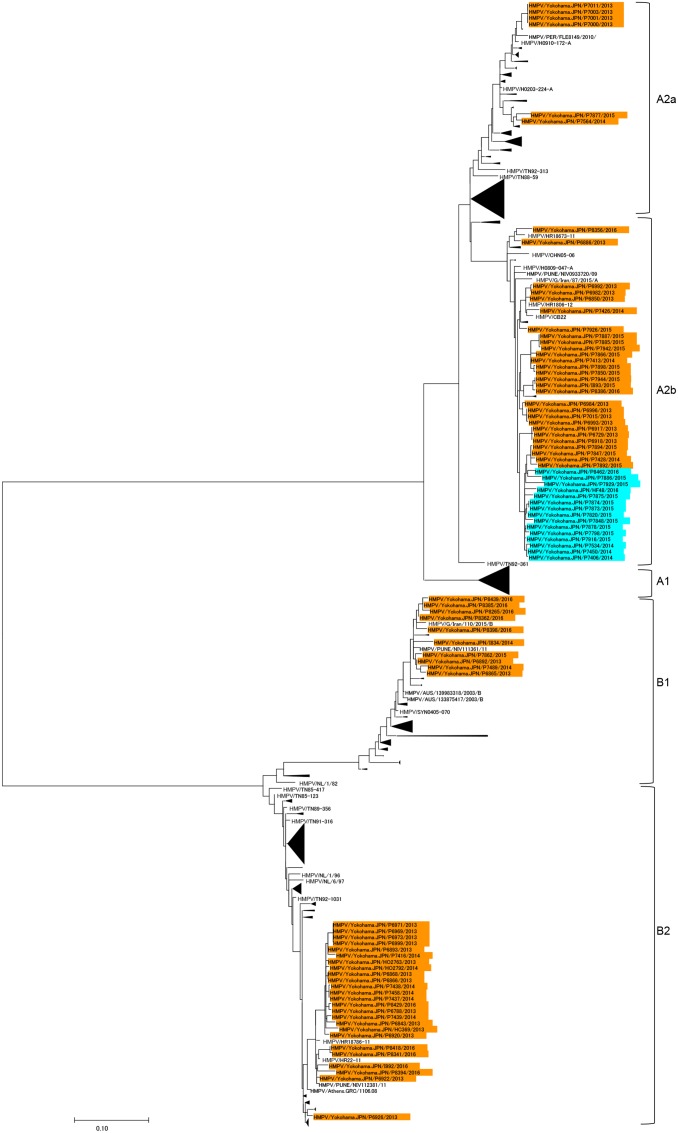
**Phylogenetic tree of 574 *Human metapneumovirus* (HMPV) strains, constructed with the maximum likelihood method.** A phylogenetic tree was constructed from the G gene sequences of 84 HMPV strains and all other available HMPV G gene sequences. In total, 574 G gene sequences of HMPV strains were included. The HMPV A2b subgroup strains containing a 180-nt duplication (180nt-dup) in the G gene (A2b_180nt-dup_ HMPV) are shown in cyan boxes and the other HMPV strains detected in Yokohama City between January 2013 and June 2016 are shown in orange boxes.

**FIGURE 2:**
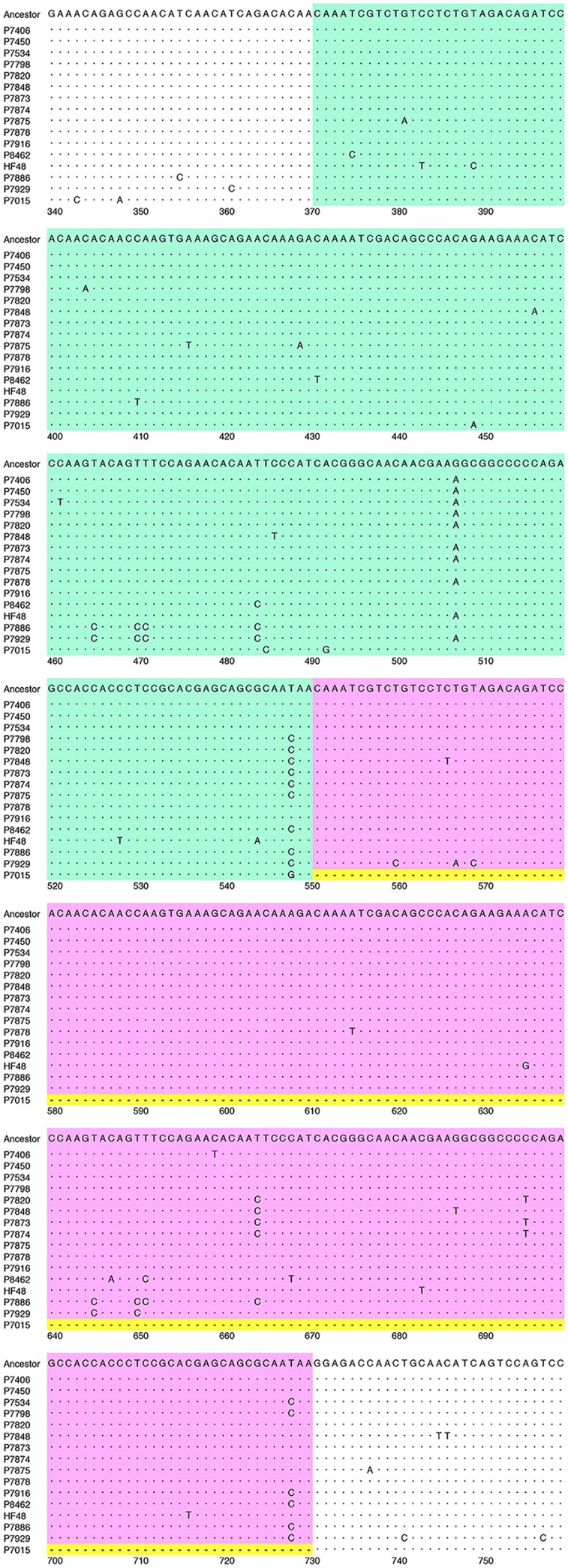
**Nucleotide sequence alignment of HMPV strains.** An alignment of the plus-sense nucleotide sequences of the 180-nt duplicated regions and adjacent regions of the HMPV G genes of 15 A2b_180nt-dup_ HMPV strains, the hypothetical ancestral strain (Ancestor), and a representative classical A2b strain (P7015) is shown. The first 180-nt region (1st-180nt) and the second 180-nt region (2nd-180nt) are shown in green and pink boxes, respectively. The first nucleotide of the initiation codon of the G gene is deemed to be nucleotide position 1. The nucleotide sequence of the hypothetical ancestral strain was predicted by constructing a consensus sequence of the duplicated 180-nt sequences of all 15 A2b_180nt-dup_ HMPV strains. Dots indicate nucleotides identical to those of the hypothetical ancestral strain. Hyphens in yellow boxes indicate gaps in the sequence.

*Human metapneumovirus* was detected in 91 of 1308 specimens from ARI patients, accounting for 7.0% of the specimens. The basic and clinical characteristics of the patients infected with HMPV are summarized in **Table 1**. The entire nucleotide sequences of the open reading frames of the G proteins were determined for 84 of the 91 HMPV strains. These 84 HMPV strains had 73 unique G gene sequences, containing at least one nucleotide substitution, whereas the remaining 11 sequences were identical to other G gene sequences. A phylogenetic analysis was performed using the G gene sequences of the 84 HMPV strains and all the HMPV G gene sequences available at the National Center for Biotechnology Information (NCBI) nucleotide sequence database^5^. In total, 574 G gene sequences of HMPV strains were used to construct the phylogenetic tree (**Figure 1**). Among our 84 strains, 6, 43, 10, and 25 strains were classified in subgroups A2a, A2b, B1, and B2, respectively (**Figure 1**). No HMPV strain belonged to subgroup A1. These data are similar to previous findings in Japan (Matsuzaki et al., 2008; Mizuta et al., 2010; Toda et al., 2010; Omura et al., 2011; Nidaira et al., 2012; Nakamura et al., 2013). **Table 2** shows the numbers of HMPV strains detected between January 2013 and June 2016 in Yokohama City and their subgroup classification. Multiple subgroup strains were detected in each year. Subgroup A2b strains were detected every year, and approximately half the A2b subgroup strains detected in 2014, 2015, and 2016 contained 180nt-dup in the G gene (**Figure 2**). 180nt-dup is a duplication of the 180 nt at nucleotide positions 371–550 (the first nucleotide of the initiation codon of the G gene is deemed to be nucleotide position 1). No A2b strain detected in 2013 contained 180nt-dup. The 15 A2b_180nt-dup_ HMPV strains formed a small cluster on the phylogenetic tree (**Figure 1**), and the cluster also contained four classical A2b strains, which lack 180nt-dup and were detected in 2014 and 2015 in Yokohama City. These data suggest that 180nt-dup occurred in a specific ancestor of the HMPV strains in this cluster (**Figure 2**).

**Table 1:** Characteristics of patients^a^ infected with *Human metapneumovirus* (HMPV) strains.

		A2a	A2b	A2b_180nt-dup_	B1	B2
Age^b^ (year)		4.8 (2.2–5.2)	3.3 (1.8–6.5)	1.8 (1.4–6.4)	2.1 (1.3–4.4)	2.6 (1.3–4.7)
Sex^c^						
	Female	3	10	4	6	7
	Male	3	14	8	3	15
Body temperature^d^		39.1 (1.3)	38.6 (2.3)	39.4 (0.7)	38.7 (1.2)	39.7 (0.7)


*^a^Of the total 84 patients, clinical information was available for 73 patients.*

*^b^Data are medians (interquartile ranges).*

*^c^Number of patients.*

*^d^Mean (standard deviation).*

**Table 2:** Numbers of HMPV strains detected in Yokohama City between January 2013 and June 2016 and their subgroup classification.

	180nt-dup^a^	Year of virus detection				Total
Subgroup		2013	2014	2015	2016	

A2a	-	4	1	1	0	6
A2b	-	11	3	12	2	28
	+	0	3	10	2	15
B1	-	2	2	1	5	10
B2	-	14	6	0	5	25
Total		31	15	24	14	84


*^a^180-nt duplication.*

### Prediction of the Year in Which 180nt-dup Occurred

**FIGURE 3:**
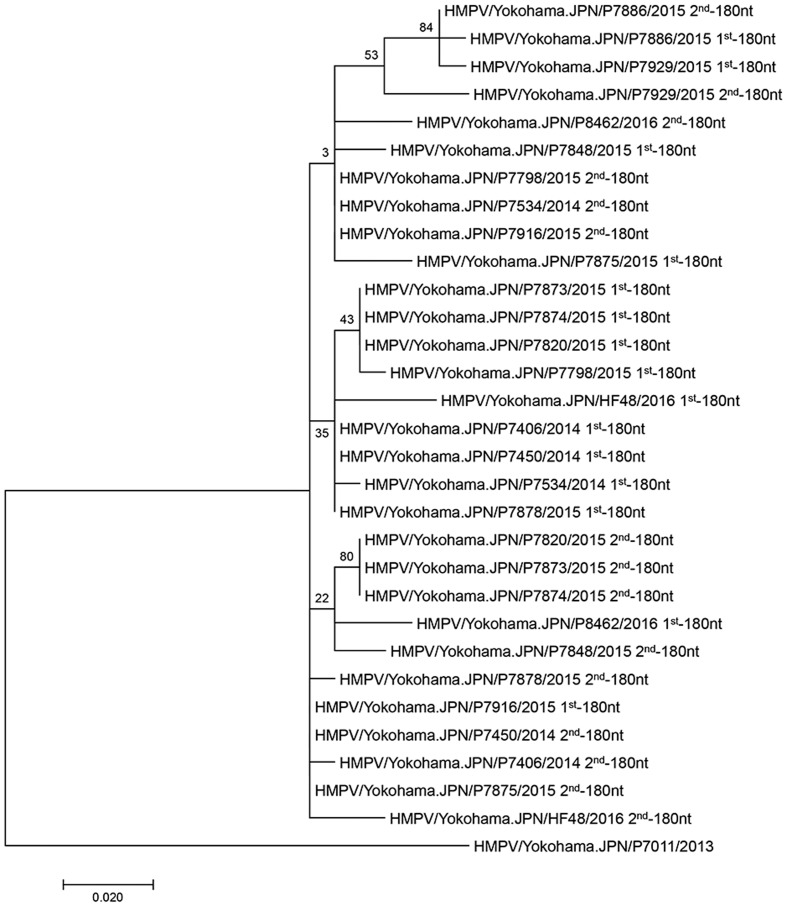
**Phylogenetic tree constructed with 1st-180nt and 2nd-180nt in the duplicated region of 15 A2b_180nt-dup_ HMPV strains.** The duplicated region contains the original 1st-180nt and the duplicated 2nd-180nt. A phylogenetic tree was constructed from the 1st- and 2nd-180nts of 15 A2b_180nt-dup_ HMPV strains, and the tree was rooted with HMPV A2a strain HMPV/Yokohama.JPN/P7011/2013. The labels at the branch nodes indicate their statistical significance, calculated with bootstrapping (50 replicates).

Although the 15 A2b_180nt-dup_ HMPV strains seemed to be derived from a common ancestor, the 180nt-dup sequences of most A2b_180nt-dup_ HMPV strains differed from one another (**Figure 2**). Even within the same viral genome, the 1st-180nt differed from the 2nd-180nt by one to six nucleotide substitutions. These observations were not unexpected, because substitutions would have occurred in each viral genome after the ancestral A2b strain had acquired 180nt-dup. RNA viruses are vulnerable to changes in their nucleotide sequences because the viral RNA-dependent RNA polymerase (RdRp) lacks proofreading activity. Therefore, the original 180-nt sequence in the hypothetical ancestral strain was predicted by constructing a consensus sequence of the 1st- and 2nd-180nts of the 15 A2b_180nt-dup_ HMPV strains (**Figure 2**). The 360-nt sequence, consisting of the 1st- and 2nd-180nts of each of the 15 A2b_180nt-dup_ HMPV strains, was then compared with that of the hypothetical ancestral strain (**Figure 2**). In the 1st-180nt, strain HMPV/Yokohama.JPN/P7916/2015 had the same nucleotide sequence as the ancestral strain, whereas the other 14 strains had one to six nucleotide substitutions relative to the sequence of the ancestral strain (**Figure 2**). In the 2nd-180nt, two strains (HMPV/Yokohama.JPN/P7450/2014 and HMPV/Yokohama.JPN/P7875/2015) had the same nucleotide sequence as the ancestral strain, whereas the other 13 strains had one to six nucleotide substitutions relative to the sequence of the ancestral strain (**Figure 2**). We then estimated the evolutionary rate of the HMPV G gene with the BEAST 2 program to predict the year in which 180nt-dup occurred. The evolutionary rate was estimated to be 4.3 × 10^-3^/site/year (95% highest probability density: 3.2–5.4 × 10^-3^/site/year), consistent with the rate previously estimated by de Graaf et al. (2008). Based on these data, 180nt-dup was predicted to have occurred in the ancestral strain between 2011 and 2013. **Figure 3** shows a phylogenetic tree constructed with the 15 1st-180nts and the 15 2nd-180nts. The tree was rooted with the HMPV A2a strain HMPV/Yokohama.JPN/P7011/2013. Theoretically, the 1st- and 2nd-180nts in each strain should be identical at the time when the duplication occurred. However, among the 15 A2b_180nt-dup_ HMPV strains, the 1st- and 2nd-180nts of 13 strains have diverged randomly and the 1st- and 2nd-180nts of each of these 13 strains were not located in the same cluster. These data suggest that these 13 strains had the same ancestral strain but acquired nucleotide substitutions in both the 1st- and 2nd-180nts independently of each other. In contrast, the 1st- and 2nd-180nts of two A2b_180nt-dup_ HMPV strains, HMPV/Yokohama.JPN/P7886/2015 and HMPV/Yokohama.JPN/P7929/2015, were located in a distinct small cluster on the phylogenetic tree, suggesting that these two A2b_180nt-dup_ HMPV strains had a different ancestral strain.

### Deduced G-Protein Amino Acid Sequences of A2b_180nt-dup_ HMPV Strains

**FIGURE 4:**
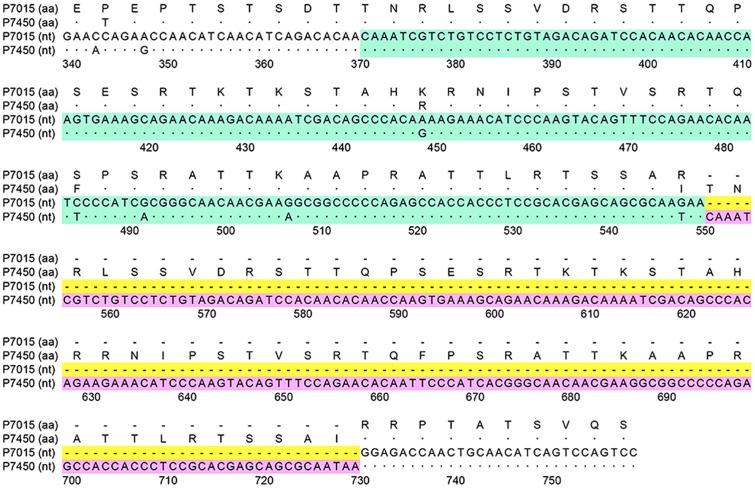
**Deduced amino acid sequence of A2b_180nt-dup_ HMPV strain.** A plus-sense nucleotide (nt) sequence alignment of a representative A2b_180nt-dup_ HMPV strain (P7450) and a classical A2b HMPV strain (P7015), together with their deduced amino acid (aa) sequences, are shown. The 1st-180nt and 2nd-180nt regions are shown in green and pink boxes, respectively. The first nucleotide of the initiation codon of the G gene is deemed to be nucleotide position 1. Dots indicate nucleotides identical to those in the hypothetical ancestral strain. Hyphens in yellow boxes indicate gaps in the sequence.

**FIGURE 5:**
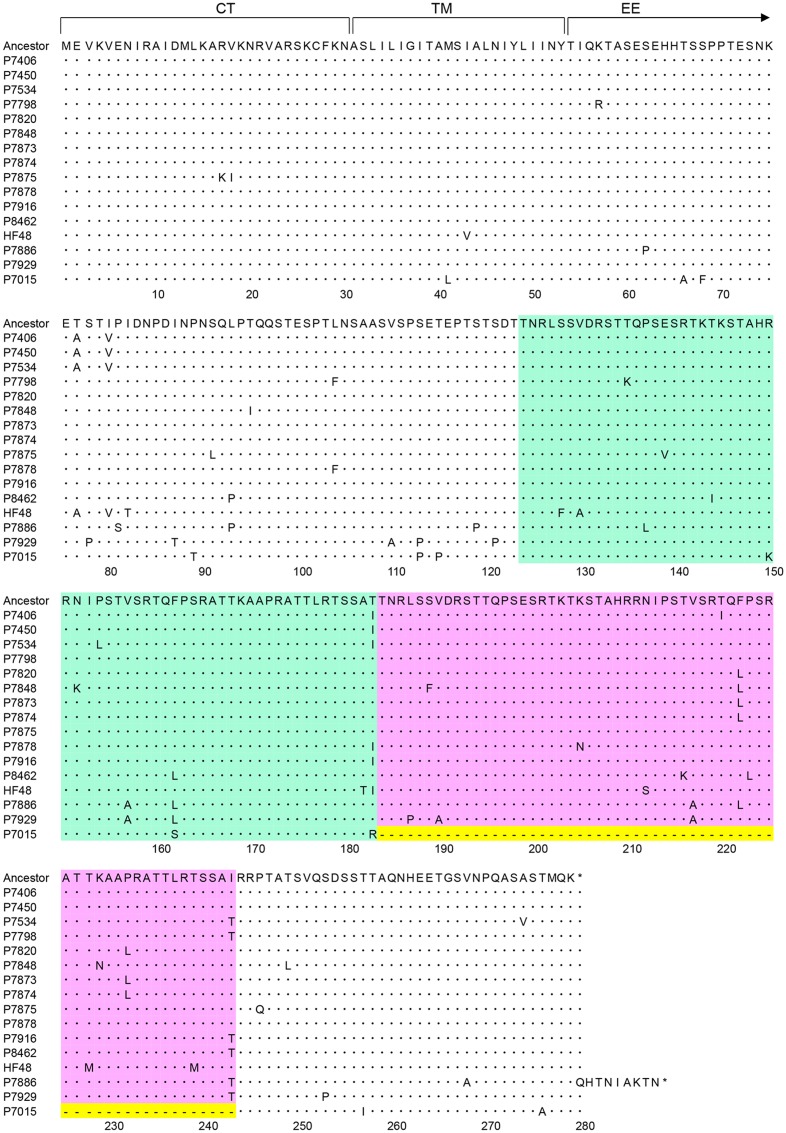
**Amino acid sequence alignment of HMPV strains.** Aligned amino acid sequences derived from the HMPV G genes of 15 A2b_180nt-dup_ HMPV strains, the hypothetical ancestral strain (Ancestor), and a representative classical A2b strain (P7015) are shown. The region between amino acid (aa) 1–30 is the cytoplasmic tail (CT) domain, and the region between aa 31–53 is the transmembrane (TM) domain. The remaining region is the extracellular ectodomain (EE). The 1st-180nt and 2nd-180nt regions are shown in green and pink boxes, respectively. The amino acid sequence of the hypothetical ancestral strain was predicted by constructing a consensus sequence of all 15 A2b_180nt-dup_ HMPV strains. Dots indicate amino acids identical to those of the hypothetical ancestral strain. Hyphens in yellow boxes indicate gaps in the sequence.

**Figure 4** shows a nucleotide sequence alignment of a representative A2b_180nt-dup_ HMPV strain (HMPV/Yokohama.JPN/P7450/2014) and a classical A2b HMPV strain (HMPV/Yokohama.JPN/P7015/2013), and their deduced amino acid sequences. **Figure 5** shows amino acid sequence alignment of the G protein of 15 A2b_180nt-dup_ HMPV strains, the hypothetical ancestral strain, and the representative classical A2b strain (HMPV/Yokohama.JPN/P7015/2013). Compared with the classical A2b strain, the A2b_180nt-dup_ strains have a 60-amino-acid duplication (60aa-dup) in the G protein. Because the 180-nt region at positions 371–550 is duplicated, a codon (AGG) for arginine encoded at nucleotide positions 550–552 is replaced with a codon (ACA) for threonine (nucleotides in the duplicated sequence are underlined; the first nucleotide of the initiation codon of the G gene is deemed to be nucleotide position 1), which is followed by the duplicated nucleotide sequence encoding 60aa-dup (**Figure 4**). The 60aa-dup is located in the C-terminal half of the extracellular ectodomain (**Figure 5**) and contains many potential acceptor sites for O-linked sugars. The G protein of the classical A2b strains, which lack 60aa-dup, have 58–63 potential acceptor sites for O-linked sugars. With 60aa-dup, the G proteins of the A2b_180nt-dup_ HMPV strains have acquired 23–25 additional potential acceptor sites for O-linked sugars. However, 60aa-dup does not affect the number of potential acceptor sites for N-linked sugars.

## Discussion

In the present study, we determined the G gene sequences of 84 HMPV strains detected in Yokohama City between January 2013 and June 2016. Our data demonstrate that HMPV strains of the four subgroups A2a, A2b, B1, and B2 were prevalent in Yokohama City in this period. Most importantly, the analysis detected 180nt-dup in the G genes of circulating HMPV A2b strains. Among the 32 A2b strains detected in Yokohama between 2014 and 2016, 15 (46.9%) contained 180nt-dup. Because 180nt-dup does not cause a frameshift in the G mRNA sequence, it generates the 60aa-dup in the G protein. A previous study (Leyrat et al., 2014) suggested that the extracellular ectodomain of the HMPV G protein sterically shields the F protein from recognition by host immune factors. These additional 60 amino acids may enhance this steric inhibition effect. The additional potential acceptor sites for O-linked sugars in the 60aa-dup region may also contribute to the evasion of immune recognition by the host, as has been observed for Ebola virus (Francica et al., 2010).

Similar duplications in the G gene have been reported in *Respiratory syncytial virus* (RSV), another member of the subfamily *Pneumovirinae* in the family *Paramyxoviridae*. A 72-nt duplication (72nt-dup) was detected in the G gene of genotype ON1 RSV strains in subgroup A (Eshaghi et al., 2012). A 60-nt duplication (60nt-dup) was also detected in the G gene of the genotype BA RSV strains in subgroup B (Trento et al., 2003). When we searched the currently available nucleotide sequence databases of viruses, we found no HMPV strain with a sequence duplication in the G gene. However, during the preparation of this manuscript, a similar report of 180nt-dup was presented at the 19th Annual Meeting of the European Society for Clinical Virology held in September 2016 (Pinana et al., 2016). Although only the abstract of the study is available, the study identified nine HMPV strains with 180nt-dup among 52 A2b strains detected in Barcelona, Spain, between 2014 and 2016 (Pinana et al., 2016). That study suggests that the virulence of the nine HMPV strains containing 180nt-dup was elevated based on the clinical manifestations of the children infected with those HMPV strains (Pinana et al., 2016). No such clinical differences were evident in our data.

Our data suggest that 180nt-dup occurred between 2011 and 2013. Importantly, these A2b_180nt-dup_ HMPV strains became some of the major strains circulating in Yokohama City within 3 years. However, further surveillance data are required to determine whether these novel HMPV A2b strains persist as predominant strains. An increased frequency of A2b_180nt-dup_ HMPV strains among the A2b strains was also observed in Barcelona, Spain (Pinana et al., 2016). Together, these data suggest that A2b_180nt-dup_ HMPV strains are already circulating globally.

Although similar duplications in the G gene have been observed in RSV, the size of the duplication in the A2b_180nt-dup_ HMPV strains is 2–3 times larger than that observed in the RSV G gene. Therefore, 60aa-dup, which results from 180nt-dup, may alter the viral antigenicity and the G protein functions more dramatically than the duplications observed in the RSV G gene. No significant difference in virulence was observed in the genotype ON1 and BA RSV strains when they acquired 72nt-dup and 60nt-dup, respectively, in their G genes (Sato et al., 2005; Tabatabai et al., 2014). However, these strains have spread rapidly and globally, and are currently the predominant strains in many countries (Trento et al., 2006; Duvvuri et al., 2015). Our data suggest that within 3 years, the A2b_180nt-dup_ HMPV strains became one of the major strains of this pathogen. The mechanism by which the A2b_180nt-dup_ HMPV strains gain an advantage over other strains when spreading in human populations, possibly by overwhelming the classical strains, and the effects of 180nt-dup in the G gene on HMPV virulence must be clarified.

## Author Contributions

MS, SU, TS, KN, and TT designed the study. MS, NN, and CK performed experiments. MS, NN, and MT analyzed data and wrote the paper. SU, TT, KN, and TS critically reviewed the manuscript.

## Conflict of Interest Statement

The authors declare that the research was conducted in the absence of any commercial or financial relationships that could be construed as a potential conflict of interest.

## Footnotes

1 http://www.cbs.dtu.dk/services/NetNGlyc/

2 http://www.cbs.dtu.dk/services/NetOGlyc/

3 http://beast2.org/

4 https://doi.org/10.1371/journal.pcbi.1003537

5 https://www.ncbi.nlm.nih.gov/nucleotide/
